# The role of the cerebellum in auditory processing using the SSI test

**DOI:** 10.1590/S1808-86942011000500008

**Published:** 2015-10-22

**Authors:** Patricia Maria Sens, Clemente Isnard Ribeiro de Almeida, Marisa Mara Neves de Souza, Josyane Borges A. Gonçalves, Luiz Claudio do Carmo

**Affiliations:** 1 PhD - FCMSCSP, Preceptor at the ENT Residency Program - Hospital do Servidor Público Municipal; 2 Professor Emeritus - Medical School of Jundiaí; Professor at the Graduate Program of the FCMSCSP; 3 Audiologist - UNIFESP. Private Practice; 4 MSc student - FCMSCSP; Assistant Professor - Federal University of Tocantins; 5 MSc in Medicine - FCMSCSP; Private Practice

**Keywords:** auditory pathways, auditory perception, cerebellum

## Abstract

**Abstract:**

The Synthetic Sentence Identification (SSI) test assesses central auditory pathways by measuring auditory and visual sensitivity and testing selective attention. Cerebellum activation in auditory attention and sensorial activity modulation have already been described. Assessing patients with cerebellar lesions alone using the SSI test can confirm the role of the cerebellum in auditory processing.

**Aim:**

To evaluate the role of the cerebellum in auditory processing in individuals with normal hearing and in those with chronic cerebellum lesions, using the SSI test.

**Materials and Methods:**

Cross-sectional cohort study. A study group comprising 18 patients with chronic cerebellar lesion and a control group of 20 healthy individuals were assessed. The SSI test was applied in an Ipsilateral Competitive Message (ICM) and Contralateral Competitive Message (CCM) modes. To compare the results between groups, we used the chi-square test for qualitative variables.

**Results:**

A statistically significant difference was found between the study and control groups using the ICM mode of the SSI test (*p*=0.035), but not in the CCM mode (*p*=0.083).

**Conclusion:**

The results on the SSI confirmed cerebellar participation in auditory processing in individuals with chronic cerebellar lesions and in those with normal hearing assessed in this study.

## INTRODUCTION

The classic idea that the cerebellum was part of the central nervous system has been changing, and numerous studies in the past 30 years have demonstrated the cerebellum participation in the cognitive process, especially that associated with hearing^1^,^2^. Despite constant investigations, there is no consensus in the literature as to how the cerebellum processes auditory information.

The studies involving auditory processing are part of an emerging set which show potential cerebellum participation in cognitive functions^1^, ^2^, ^3^, ^4^, ^5^, ^6^, ^7^, ^8^. The cerebellum connection with auditory areas started to be investigated before the main studies associating cerebellum and cognition. Snider & Stowell, in 194^4^,^9^, were the pioneers in identifying cerebellar auditory areas in cats by means of evoked auditory potentials. Many other studies were carried out afterwards using acoustic and electric stimuli in different species of animals, establishing the neuroanatomical basis which associated the cerebellum with hearing. The animal models enabled researchers to outline the pathways which connect the cerebellum to other brain structures, helping one understand the cerebellum position in the complex neuron networks regulating cognitive functions^9^, ^10^, ^11^.

With the new functional neuroimaging exams, studies started to be carried out in human beings, trying to demonstrate which the cerebellum participation in cognition is. These studies provided surprising evidence that the cerebellum is intensively and selectively active during sensorial and cognitive tasks, even in the absence of implicit or explicit motor behavior^1^, ^2^, ^3^,^6^,^12^,^13^.

Knowledge about auditory pathway processing is not totally clear. Auditory processing involves not only the perception of sounds and their integration with other types of sensitivity, but also how they are identified, located, submitted to attention, analyzed, memorized and recovered. Auditory processing may be associated with difficulties in hearing, speech understanding, language development and learning^14^.

The investigation of the relationship between the cerebellum and the auditory pathways has produced a substantial body of evidence which requires the review of concepts about its functional role, including informing patients and family members about the need to understand the behavioral consequences of cerebellum diseases and rehabilitation treatments.

The Synthetic Sentences Identification test (SSI) is an important means of diagnosis and easy to perform in the assessment of central auditory pathways, using auditory and visual skills. The physiological mechanism assessed in the test is associated with the inhibition of sounds which, although present in the communication environment, are relatively ignored, and this may be considered selective attention. Numerous studies have shown the cerebellum activation during auditory attention tasks, as well as its role in sensorial activity. Therefore, assessing patients with cerebellar lesion by means of the SSI test may confirm or refuse the hypothesis of cerebellum participation in auditory processing.

The SSI test may also provide information as to the supra or infratentorial location of the lesions; and in the ICM mode, test faults happen in infratentorial disorders or lesions. As to the affected side, the lesions cause changes to contralateral stimuli responses^15^, ^16^, ^17^, ^18^. With these characteristics, this test may be used as a tool to prove the association between the cerebellum and auditory processing, and there is the need to carry out further studies associating SSI test findings in individuals with cerebellum lesions.

The goal of the present study is to use the SSI test to assess cerebellum participation in the auditory processing of individuals with chronic cerebellar disorders and normal tonal audiometry.

## MATERIALS AND METHODS

Our sample had 20 healthy individuals making up the control group and 18 individuals with chronic cerebellar lesions, making up the study group. Because of the very nature of the affliction, the study group was sequentially allocated. Both groups were made up by individuals from both genders, with ages varying between 9 and 56 years, without distinction of race and without a past of ear disease. All individuals signed an informed consent form after proper explanations from the researcher. This research project (376/05) was submitted to and approved by the Ethics in Research Committee of the institution and registered at SISNEP/MS under number CAAE 0009.0.270.000-05.

Inclusion criteria: no ear diseases, no exposure to noise, no use of ototoxic drugs and no family history of hearing disorder. Individuals with hearing thresholds in the frequencies of 250 to 8000 Hz within normal values (up to 25 dB HL); type A tympanometry (pressure peak between +100 and -100 daPa), alphabetized and able to read easily. For the study group, the individuals had clinically diagnosed chronic cerebellar lesion, confirmed by an image study, without evidence of central nervous system lesions outside the cerebellum.

Exclusion criteria: individuals with visual disorders which prevented them from reading the phrases and individuals with important motor incoordination.

## METHODS

In both groups we assessed auditory threshold values in the frequencies between 250 and 8000 Hz, vocal discrimination, including the speech recognition threshold and the speech discrimination index, with an Itera audiometer (Madsen^®^). Tympanometric curve with the Madsen® ZS77-MB device.

The SSI test was carried out in a sound treated booth, with the phonetic message being introduced through earphones. The message was reproduced by recording the phrases and a competitive message was provided, by a two-channel Itera audiometer (Madsen^®^). A list with 10 third-order synthetic phrases with seven words each (phrases presenting three words with meaning and dependence among each other), without semantic meaning, was transmitted to the patient, who pressed the button corresponding to the phrase heard after reading a printed set of these phrases located in front of him/her. Two series of third-order phrases were employed in both groups at a fixed intensity of 40dB SL, having as basis the mean values of the air conduction tonal auditory thresholds in the frequencies of 500; 1,000 and 2,000 Hz. The first series was introduced as an ipsilateral competitive message (ICM), in the phrases/competition ratio 0 and -10 dB, in other words, the competitive message was introduced in the same intensity as the phrases and at 10 dB above the intensity of the phrases and the other with the phrases being introduced in one ear and the competitive message in the contralateral ear (CCM), in the phrases/competition ratio 0 and -40 dB. Ten sentences were presented for each phrases/competition ratio to both ears. The test was first presented to the right ear and then to the left. The correct answers were analyzed in terms of their percentage, thus the performance of the individual was established by the percentage of correct answers in the different test situations.

We considered normal those results equal to or higher than 80% of correct answers for the ICM mode in the phrase/competition ratio of 0, and results equal to or higher than 70% of correct answers for the phrase/ competition ratio of -10. In the CCM mode, we considered normal those results equal to 100% of correct answers in the phrase/competition ratio of 0, and results equal to or higher than 90% of correct answers for the phrase/ competition -40, according to normal values listed in the national and international literature^17^,^19^.

In order to compare the results between the groups, we used the chi-square test for qualitative variables. The software used was the Epi Info^®^ version 3.4. The significance level (α) was fixed at 5% (< 0.05).

## RESULTS

In the study group, age varied between 9 and 56 years, seven women and 11 men, and statistically significant differences were not found insofar as gender and age were concerned between the study and control groups. The time of cerebellar lesion evolution varied between two months and seven years. As to its location, six were on the left side and six were bilateral. In relation to its etiology, nine individuals had tumors; three had stroke sequela, three had congenital changes and three individuals had cerebellar ataxia.

As to the SSI results in the ICM and CCM modes in the control group, we observed two individuals with an altered response in the ICM mode and no alterations in the CCM mode.

**Graph 1:**
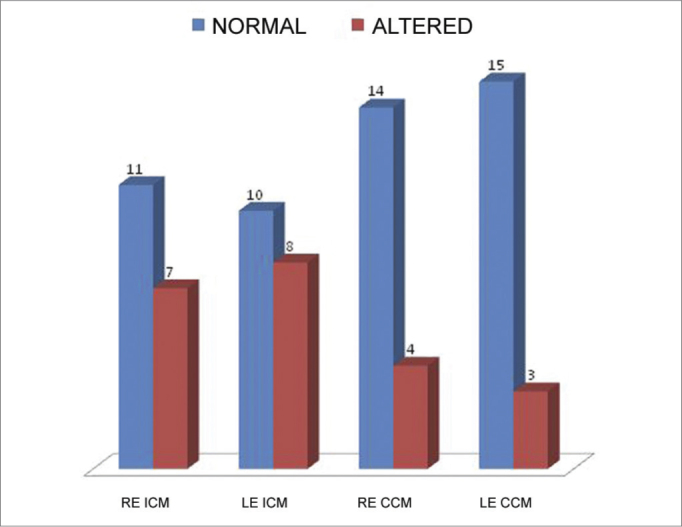
Results from the SSI test in the ICM and CCM modes in the Study Group.

In the ICM mode of the SSI test, eight individuals had altered responses in the study group, seven with changes to both ears and one with a unilateral change. In the ICM mode of the SSI test, eight individuals had changed responses in the study group, seven with changes in both ears and one with a unilateral change. As far as the CCM mode is concerned, we observed three cases of altered response, three bilateral and one unilateral (Graph 1).

In the control group, two individuals had changed results in the ICM mode of the test, one unilateral and another being bilateral; while in the study group, eight individuals had a changed result, most bilaterally. The comparison between the control and study groups presented a statistically significant difference, *p*= 0.035 (Table 1).

**Table 1:** Distribution of the Control and Study Groups, in absolute (n) and relative (%) values in relation to a disorder in the SSI test with ICM in the intensity between 0 and -10 dB.

Group		ICM involvement			Total
		Normal	Unilateral	Bilateral	
Control	n (%)	18(90.0)	1(5.0)	1(5.0)	20(100.0)
Study	n (%)	10(55.6)	1(5.6)	7(38.9)	18(100.0)
Total	n (%)	28(73.7)	2(5.3)	8(21.1)	38(100.0)

*p* = 0.035 (Chi-square).

In the SSI test, CCM mode, there were no changes in the control group. In the study group, there was one unilateral and three bilateral involvements; however, without statistically significant differences between the groups, *p*= 0.083 (Table 2). The three bilateral cases had concomitant changes in the ICM mode of the test.

**Table 2:** Distribution of the control and study groups, in absolute (n) and relative (%) values, in relation to a disorder in the SSI test with CCM in the intensity between 0 and -40 dB.

Group		Involvement CCM			Total
		Normal	Unilateral	Bilateral	
Control	n (%)	20 (100.0)	0 (0.0)	0 (0.0)	20(100.0)
Study	n (%)	14 (77.8)	1 (5.6)	3 (16.7)	18(100.0)
Total	n (%)	34(89.5)	1 (2.6)	3 (7.9)	38(100.0)

*p* = 0.083 (Chi-square).

## DISCUSSION

We did not find studies involving the SSI test and individuals with cerebellar lesions in our review of the literature; thus, it was not possible to compare our results with those from other studies. Therefore, the present paper is original in relation to the literature reviewed.

The adaptation of the SSI test into Portuguese showed results which were similar to those of the original instrument, in English; and this validates the test to be employed in this study^14^. In standardizing the SSI test for the Portuguese language, the authors noticed that the test performance in the normal population is within normal ranges, with a reliability rate of 75%^19^. This information was also seen in our Control Group.

Although there are fancy tests, i.e. functional neuroimaging, used to study cognitive changes, the SSI has the advantage of being extremely simple, allowing its clinical application not only to identify the deficiency, but also in the follow up of the patient's recovery.

Studies have reported that the SSI test helps identify intra-axial lesions in the brainstem, and those individuals with such lesions have a worse performance in the monaural mode of the test (ICM)^15^,^16^. Later studies have reported on the lower performance of patients with supratentorial lesions in the binaural mode of the test (CCM), extending the SSI test use to differentiate lesions in distinct structures (infra and supratentorial regions)^17^,^18^. This data was useful for the present paper in order to interpret the qualitative assessment, where we found a statistically significant difference in comparing the responses from the control and study groups in the ICM mode, in the phrases/competition ratios between 0 and -10 dB (Table 1). Thus, this test enabled us to identify those individuals with central infratentorial auditory pathway - cerebellum - disorders, confirming the topographic diagnosis of the auditory pathway lesion.

Another interesting application for the SSI test in individuals with cerebellar disease would be to follow them up during rehabilitation programs, when one could observe the individual's performance in the test as the cerebellar function is corrected^14^. This performance assessment using cognitive tests has been mentioned in another study, which identified a lower number of errors in the cognitive test done months after the surgery to remove the cerebellar lesion^4^.

Since the SSI test is able to assess each side separately, we detected that in those individuals from the study group in the ICM mode who had altered results, most happened bilaterally (Graph 1). Nonetheless, studies which assess infratentorial lesions found changes in the SSI test in the ICM mode ipsilateral to the infratentorial lesion^15^,^16^,^18^. Such information alerts us about a possible extensive involvement of the auditory processing in individuals with cerebellar lesion, affecting the individual's entire performance.

In the literature we found a predominance of rightside cerebellar activation in response to the auditory stimuli in normal individuals, followed by the bilateral cerebellar activation^3^,^6^,^13^. And, moreover, individuals with bilateral cerebellar lesions, had deterioration in their perception of temporal aspects associated with speech^5^, while individuals with unilateral lesions had similar results to those from the control group. In another study, individuals with right cerebellar lesion had changes in their verbal performance, fluency and auditory sequential memory. Those with lesions in their left cerebellum, had changes in their non-verbal performance and in their visual sequential memory^7^. Evidence of the reduction of the phonologic similarity effect in individuals with right cerebellar lesion was observed when compared to those who had a lesion on the left side^20^. The present study also showed that individuals with right cerebellar lesion tended to have altered results in the SSI test, but without statistically significance differences - because of the small sample. The SSI test does not allow for this qualitative analysis in relation to all the modes of auditory processing alterations, but it allows one to identify those cases in which other, more sophisticated, tests must be employed.

In the SSI test we used the integration between the auditory and visual stimuli, since the individual had to identify the phrase heard among ten written in front of him/her. This integration of sensorial stimuli has been reported in numerous papers about the cerebellum. Studies have suggested a common neural network for the visual and auditory memories, and for the auditory and visual processing of words, respectively, involving the left-lower, anterior-pre-frontal, supplementary-anterior motor, upper parietal cortex and posterior cerebellar areas^3^,^6^. Studies assessing the auditory and visual memories of children with cerebellar lesions have identified changes in the tests with both stimuli^4^. This information place the cerebellum as a convergence area for sensorial stimuli, connecting it with supratentorial areas by afferent and efferent pathways. This knowledge explains the changes seen in the CCM mode of the test which captures supratentorial dysfunctions, without evidence of supratentorial lesions in the cases studied under image exams, but it is likely that the cerebellar lesion severely affected these pathways, since three of the four altered cases in the CCM mode had all the other ICM mode tests altered and only one in the CCM mode had normal ICM.

The activation of different cerebellar areas leads us to infer about a functional specificity of cerebellar areas, suggesting an analogy with computer functions, in which the cerebellum behaves as a computer which processes specialized data, integrating information from numerous origins and modes^8^.

The great difficulty in finding patients with cerebellar lesions only made our study group very restrictive, including individuals with different cerebellar lesion etiologies. Further studies are needed, with a greater number of individuals, in order to have a better understanding of the functional role performed by the cerebellum.

## CONCLUSION

The results obtained by the SSI test pointed to the cerebellum participation in auditory processing in individuals with chronic cerebellar lesions and normal tonal hearing assessed in this study.
